# Does crystallography need a new name?

**DOI:** 10.1107/S2052252517009484

**Published:** 2017-06-29

**Authors:** Dimitri Argyriou

**Affiliations:** a Ames Laboratory, Iowa, 50011, USA

**Keywords:** editorial, crystallography

## Abstract

Our notion of crystallography and how we communicate it needs to adapt to the major emerging frontiers of science in order to ensure that our community continues to thrive.

The discovery of X-rays and their use in the observation of diffraction from crystals placed crystallography at the forefront of science at the beginning of the last century. The combination of this new tool, together with the emerging understanding of the symmetry of crystals, exposed the locations of atoms in matter and allowed us to start understanding macroscopic properties from an atomic perspective for the first time. These discoveries transformed physics and chemistry bringing to light new scientific fields such as materials science and structural biology.

There is no doubt that the field of crystallography is blossoming as much as it did at the start of the last century. Today, our science has moved to be far more than crystal structures (Larsen, 2015 ▸). Cryo-electron microscopy and free-electron lasers have revolutionized the way we do and think about crystallography, allowing us to tackle with relative ease complex biological structures or to take crystallography to unthinkably small time frames and nanocrystals.

Despite these success, university departments are becoming all too reluctant to invest in chairs or professorships in crystallography. Traditional chairs in crystallography are disappearing and are increasingly replaced by far more topical and palatable sounding names. Irrespective of whether we are comfortable with this trend, the name ‘Crystallography’ conjures up images of an old, last century science that has essentially no new frontiers left to tackle despite its amazing successes that are continually transforming science. As it has been pointed out previously in these pages (Hasnain, 2015 ▸), part of this phenomenon is crystallography’s own success. Crystallography is a precise science that, with modern technology, is amenable to an enormous degree of automation, even to the extent of making automated measurements, analysis and submitting the results to databases and journals!

The consequences of this trend are troubling. Intuitively, and looking at the demands of science, we have, more than ever before, an increasing need for scientists with excellent training and understanding of symmetry in crystals. With crystallography trending away from the spotlight of modern science, it is unclear where this training will come from. In the meantime, the codes that power much of the work that we do are challenged by personal succession issues, creating emergencies in not only developing new or maintaining existing codes, but even in understanding what is in the old *FORTRAN* codes that we use today.

This worrying perception regarding crystallography is in part an acceptance of its wide impact in science and at the same time a failure to recognize its transformation and colonization of many different fields of science. It is wonderful that the frontiers of science are shifting but we also need to shift with them. While the symmetry of the crystalline state is of vital interest, we have arrived at a point well beyond our dreams of manipulating atoms to place them where we wish to achieve the function we desire. In biology, we are able to engineer and manipulate proteins or even edit DNA sequences, while in physics we use crystallography to understand and manipulate novel electronic states in emerging topological quantum materials. Indeed, the beautiful science of topological insulators (Cava* et al.*, 2013 ▸) and multiferroics (Radaelli & Chapin, 2007 ▸) at the electronic level is based on symmetry arguments where crystallography provides the proper context. The diverse impact that crystallography is having is plainly evident in the pages of **IUCrJ**. Crystallography sheds insights into materials as diverse as metallic nanocrystals, thin films and polymers (Peterson & Papadakis, 2015 ▸), colloids (Sandy, 2014 ▸) or the development of new wide-band-gap semiconductors (Klimm, 2014 ▸). Crystallography and crystallographers have always been inclusive and the challenge ahead lies in what we define as crystallography going forward into the future.

If we accept that crystallography has actually progressed significantly but under the guise of different names, should we then ask the question – does crystallography need a new name?

In part, this has been happening already. ‘Structural Biology’ and ‘Physical Crystallography’ are two of the descriptions that have surfaced in recent years, both recognizing crystallography as a tool at the center of understanding a broader field. Indeed, what some of the recent papers in **IUCrJ** and related journals have highlighted goes beyond describing the location of atoms but rather uses crystallography as the basis to explore the symmetry of other states, often electronic in matter.

Clearly, our notion of crystallography and how we communicate it needs to adapt to the major emerging frontiers of science in order to ensure that our community continues to thrive. Already IUCr journals are broadening their horizons but more needs to be done at many levels to define a broader and more inclusive agenda for modern crystallography.

This to some may be simply a marketing challenge, but the real issue here is a recognition that crystallography is essential and university departments must continue to be persuaded to invest in, and to train, the next generation of crystallographers.
